# JIB.tools 2.0 – A Bioinformatics Registry for Journal Published Tools with Interoperability to bio.tools

**DOI:** 10.1515/jib-2019-0059

**Published:** 2020-01-08

**Authors:** Marcel Friedrichs, Alban Shoshi, Piotr Jaroslaw Chmura, Jon Ison, Veit Schwämmle, Falk Schreiber, Ralf Hofestädt, Bjorn Sommer

**Affiliations:** Bielefeld University, Faculty of Technology, Bioinformatics/Medical Informatics Department, Bielefeld, Germany; University of Copenhagen, Center for Protein Research, Copenhagen, Denmark; Technical University of Denmark, Department of Bio and Health Informatics, Lyngby, Denmark; University of Southern Denmark, Department of Biochemistry and Molecular Biology, Protein Research Group, Odense, Denmark; Konstanz University, Life Science Informatics, Konstanz, Germany; Royal College of Art, School of Design, London, UK

**Keywords:** Computational Biology, Databases as Topic, Collection

## Abstract

JIB.tools 2.0 is a new approach to more closely embed the curation process in the publication process. This website hosts the tools, software applications, databases and workflow systems published in the Journal of Integrative Bioinformatics (JIB). As soon as a new tool-related publication is published in JIB, the tool is posted to JIB.tools and can afterwards be easily transferred to bio.tools, a large information repository of software tools, databases and services for bioinformatics and the life sciences. In this way, an easily-accessible list of tools is provided which were published in JIB a well as status information regarding the underlying service. With newer registries like bio.tools providing these information on a bigger scale, JIB.tools 2.0 closes the gap between journal publications and registry publication. (Reference: https://jib.tools).

## 1 Introduction

JIB.tools is a new approach to present tools, services, databases, and workflow systems, which were published in the Journal of Integrative Bioinformatics (JIB), in a public, web-based registry.

JIB is a long-running journal launched in 2004 as the official journal of the IMBio association (today: “FG Informationsmanagement in der Biotechnologie of the German Society of Computer Science”). This journal is focused on software applications/tools and databases covering a wide range of topics, for example: Molecular Databases, Information Systems and Data Warehouses, Data Integration, Methods and Tools, Metabolic and Regulatory Network Modeling, Network Analysis, Text Mining, Integrative and molecular modeling; as well as Visualization and animation.

With a large number of bioinformatics tools available and new ones constantly being developed, finding the right tool for a certain task can be problematic. The bio.tools registry alone currently lists over 12.000 tool entries [1]. Therefore, listing and annotating bioinformatics tools and databases with metadata is an important task. Keyword annotations like topics for which kind of tasks the tool is intended help reduce the search space and on the other hand increase the visibility of tools in their respective topic.

### 1.1 JIBtools 1.0

JIBtools 1.0 was officially released in 2013 [2]. The initial version had a different concept than JIBtools 2.0: it was a curator-based system. It contains the basic introduction as well as the different Tool Lists with the associated editors. The basic idea was that the editor of each section curates the list once a year to extend it with new tools and remove those ones which are not online anymore. In theory, this was an eligible approach, but in practice, nobody had the time to curate these lists manually.

A Tool List – e.g. “3D Network Modeling and Visualization” – contained a list of related tools with the corresponding name, the link, the publication, as well as the abstract. Such a Tool List contained tools published in JIB as well as those ones published elsewhere, but still relevant for the category. Moreover, different categories were used, represented as simple icons – here, 2.5D (for 2D networks stacked in 3D space, such as [3]) and 3D (for 3D networks, such as in [4]).

The framework of JIBtools was based on the phpBB forum [5]. phpBB is a free and customizable forum bulletin board software solution which is widely used in the Internet and which offers many different styles, extensions and templates (https://www.phpbb.com). It was customized to be used by one main administrator and a number of co-administrators – the editors. The editors had only access to their own Tool List. The Tool entries were based on a template which the users had to post into their forum for each tool.

The problem of this approach was that the editors did not have a strong intrinsic motivation to curate their tool lists. With the initial launch of JIBtools 1.0 in 2013 and the last entry curated in december of the same year, the tool list quickly became outdated. Related approaches nowadays give the users the opportunity to post their own tools to the tool lists and promote their own work.

For more information, please see the Supplement: Figure S1 shows the main page of JIBtools 1.0, and Figure S2 shows the Tool List of “3D Network Modeling and Visualization”.

## 2 Related Work

In parallel to JIBtools 1.0, multiple registries for bioinformatics and visualisation software were made available online with similar goals. Some of these resources have the aim to provide an exhaustive list of tools in a standardized way. In contrast to JIBtools, registries like bio.tools had great success with the help of a community of authors and curators and quickly surpassed the capabilities of JIBtools.

Here, different registries are being analyzed to find a niche in which the new version of JIBtools can improve tools representation and interconnecting with other registries.

### 2.1 bio.tools

bio.tools is a comprehensive registry of tools and data resources for life sciences built as a part of the ELIXIR’s (European Infrastructure for Biological Information) Tools Platform [6], [1]. The registry uses the EDAM Ontology [7] for precise tool descriptions, making it easier for people to find, understand and compare software tools with respect to their scientific

Tools Platform [6], [1]. The registry uses the EDAM Ontology [7] for precise tool descriptions, making it easier for people to find, understand and compare software tools with respect to their scientific topics, operations, types of data and data formats. bio.tools offers a Graphical User Interface in the form of a web portal and a documented API intended to be used by service providers. The entries in bio.tools are annotated with publications and related scientific artifacts through use of widely accepted identifiers (e.g. PMID, DOI) that will allow JIBtools to extend its content by integrating information from external sources.

### 2.2 Atlas of Biological Databases and Tools

The Atlas of Biological Databases and Tools (DaTo) is a data-mining approach to the problem of finding and listing bioinformatics tools and databases [8]. All abstracts from PubMed are computationally analyzed for a URL and raw information, such as names and description. A world map of host locations for all tools in the registry is provided on the DaTo website and the status of the tools URLs visualizes their availability. The elegant idea to use text-mining for data integration and very comprehensible world map are overshadowed by problems such as malformed URLs and the overall lack of more detailed and curated information.

### 2.3 Biological Visualisation Network

The Biological Visualisation Network (BiVi) is an online community for life-scientists to discover and promote complex data visualisation ideas and solutions [9]. The visualisation tool registry is manually curated and developers are able to register and add new tools. Entries can be annotated with tags for the used technology, platform, availability, and many more. Additionally, the developers are able to add a description, web links to the homepage and source code, related publications, and the status of the project. Fitting the theme of visualisation tools, screenshots of the tool can be uploaded and displayed on the registry overview and tool details page.

### 2.4 MyBioSoftware

MyBioSoftware is, in contrast to the purpose-built registry solutions bio.tools, DaTo, and BiVi, a WordPress-based blog [10]. Each blog post represents a bioinformatics software with curated information. New entries are manually added irregularly and suggestions can be made via an email to the editor of MyBioSoftware. Tool information include the name, description, publications, download link, and more. All posts are tagged with keywords like “Omics” or “Microbiology”. Many tool entries are also enriched with multiple screenshots.

### 2.5 Workflow Publishing Sites

In contrast to previous tools and visualization registries several online registries exist where users can publish scientific workflows like the Galaxy project [11], or the KNIME workflow hub [12]. These workflows operate on a higher level than single bioinformatics tools, defining specific or general executions of multiple tools or scripts in order to process or generate new insights. Therefore, these workflow sites have a different focus than JIBtools and previously mentioned registries. Both approaches are important for tool and scientific workflow visiblity and complement each other. For example, tool registries can provide an overview of available software in the development of new workflows.

### 2.6 Summary

**Table 1:** The table shows an overview of the different tool lists discussed here (Reference Date: 21.10.2019).

**Category**	**bio.tools**	**BiVi**	**DaTo**	**MyBio Software**	**JIBtools 1.0**	**JIB.tools 2.0**
Last update	2019	2019	2016	2019	2013	2019
Entries	12.971	176	17.168	12.489	122	102
Fully automatized			✓			
Semi automatized	✓					✓
Author editing	✓	✓				✓
Editor-curated	✓			✓	✓	✓
Engine	Open source	Proprietary	Proprietary	WordPress	phpBB	Proprietary
URL	[13]	[14]	[15]	[16]	[17]	[18]

Table 1 shows an overview of the different tool lists discussed here.

bio.tools contains a comprehensible number of tools and is supported by ELIXIR, therefore the outreach of this resource is quite huge. Also, a big advantage is the fact that the tool entries are editable by authors. DaTo automatically integrates tools based on text mining of abstracts, but it is not accessible by external authors. It also does not provide a categorized overview of web tools. However, based on numbers DaTo is the leading resource in our comparison and is a very interesting tool in terms of geographical visualization of tool developments. MyBioSoftware contains also an impressive large amount of tools, especially given the fact that the tool list is curated by one editor. Tool authors can make suggestions, but it is not possible to edit entries. Whereas BiVi is relatively small as it focuses on bio visualization-related topics, the presentation of the tools looks more appealing than the one of bio.tools as screenshots and images are provided. The editor-curated JIBtools 1.0 provides only a very small number of tools, given that the focus was quite broad. For each of the five approaches, the entry barrier is quite high: authors have to be motivated to submit their tool entries, or in case of DaTo, the approach is automatized, but then the authors cannot edit their entry. With JIB.tools 2.0 we are trying to provide an approach which ingests basic tool information right from the start and then motivates authors to update and improve their entries. Looking at Table 1 it is obvious that JIBtools 2.0 contains the smallest number of entries in our comparison, but the reason is that only tools and databases published in JIB are listed here. In comparison to the tools listed in bio.tools, this is roughly 0.8%, which is for a single journal quite good.

## 3 Implementation

JIB.tools 2.0 is a complete re-implementation based on a standard web-technology stack with Bootstrap 4 [19] and an SQL database backend. The goal is to provide a custom built and comprehensible user interface (UI) for visitors and curators alike. Bootstrap is a widely used, open source UI framework for the web and was chosen for the simplicity in setup and ability to quickly build a beautiful and easy-to-use frontend for JIB.tools. While there are many different UI frameworks available online, Bootstrap has already been used successfully in previous projects.

**Figure 1:**
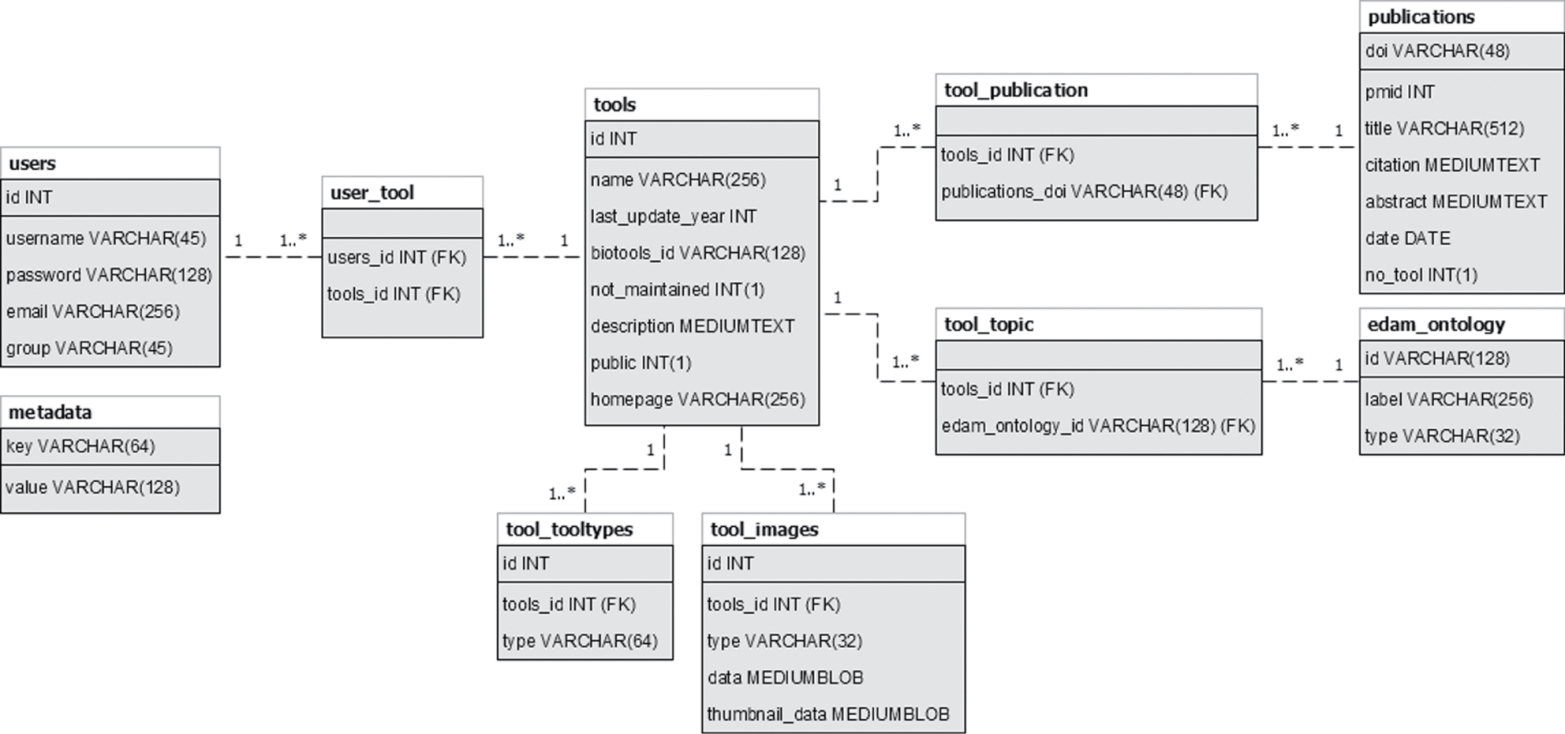
Database schema for JIB.tools 2.0 with the main entities tool, publication, and user. Additional information like types, topics, and images are linked via foreign keys to their respective entities.

**Figure 2:**
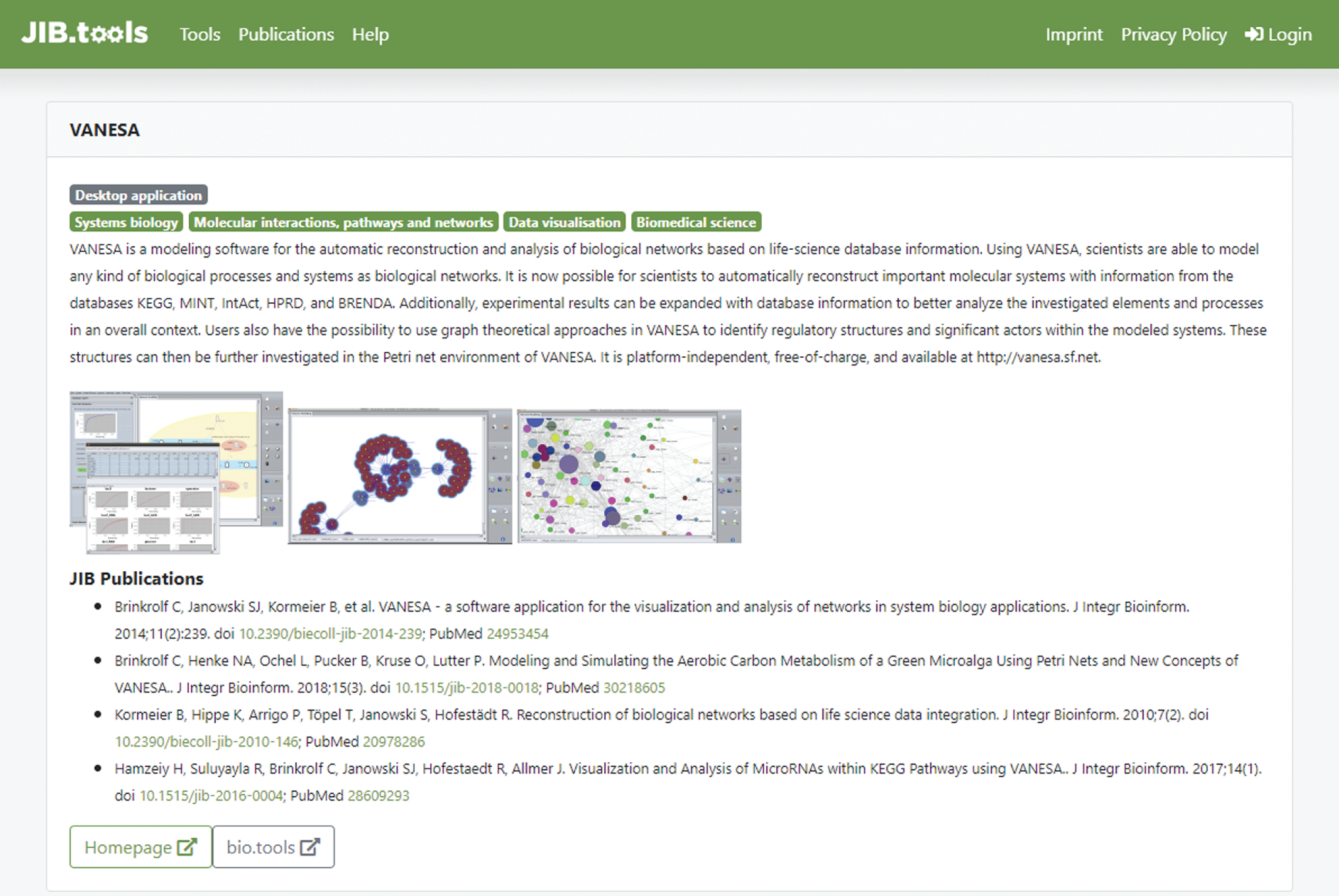
JIB.tools 2.0: details page for the “VANESA” tool published in JIB. It shows the use of tags synchronized from bio.tools, references of JIB publications, three screenshots, and other information.

**Figure 3:**
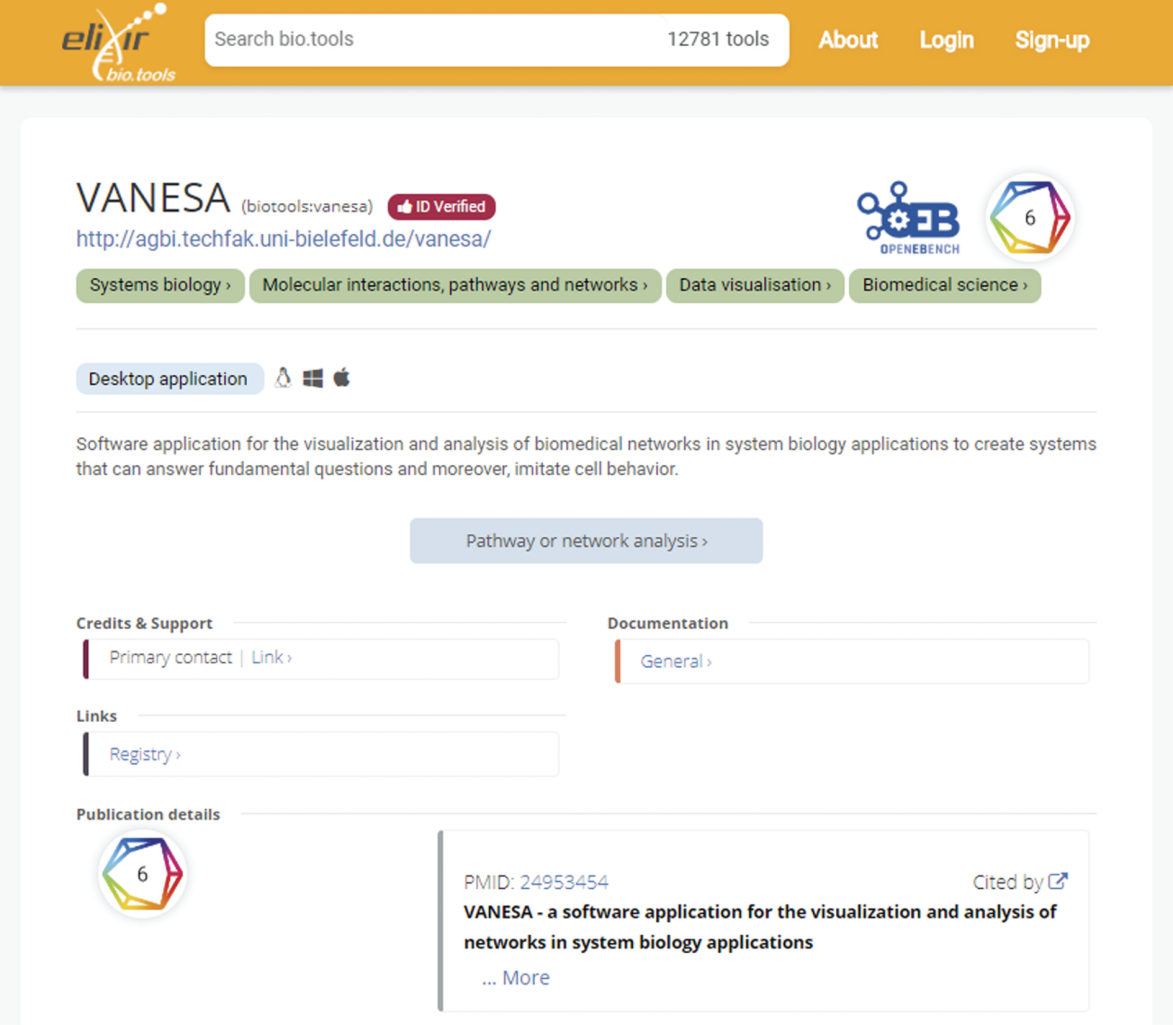
Details page for the “VANESA” tool published in the bio.tools registry.

JIBtools 1.0 – based on the phpBB forum framework – only provided limited fields for each tool in a thread tree-like structure for topic categories. The new database schema specifically captures the concept of a tool being mentioned in one or potentially multiple JIB publications. Metadata fields were selected for interoperability with bio.tools, like tool name, description, homepage link and more. The complete database schema is shown in Figure 1. Tools can be annotated with bioinformatics topic keywords using the EDAM ontology and type keywords such as “Desktop application” or “Command-line tool” matching the topics and types used in bio.tools [7]. These keywords are added by autocomplete input fields to support a fast and error-free annotation. The main difference to the bio.tools matching fields are screenshot images that can be uploaded for every tool and stored directly in the SQL database schema. Tools are limited to three images with a maximum size of 800x600 pixels and automatically cropped if bigger. An example details page for the “VANESA” tool published in JIB is shown in Figure 2. The equivalent entry in the bio.tools registry is shown in Figure 3.

**Figure 4:**
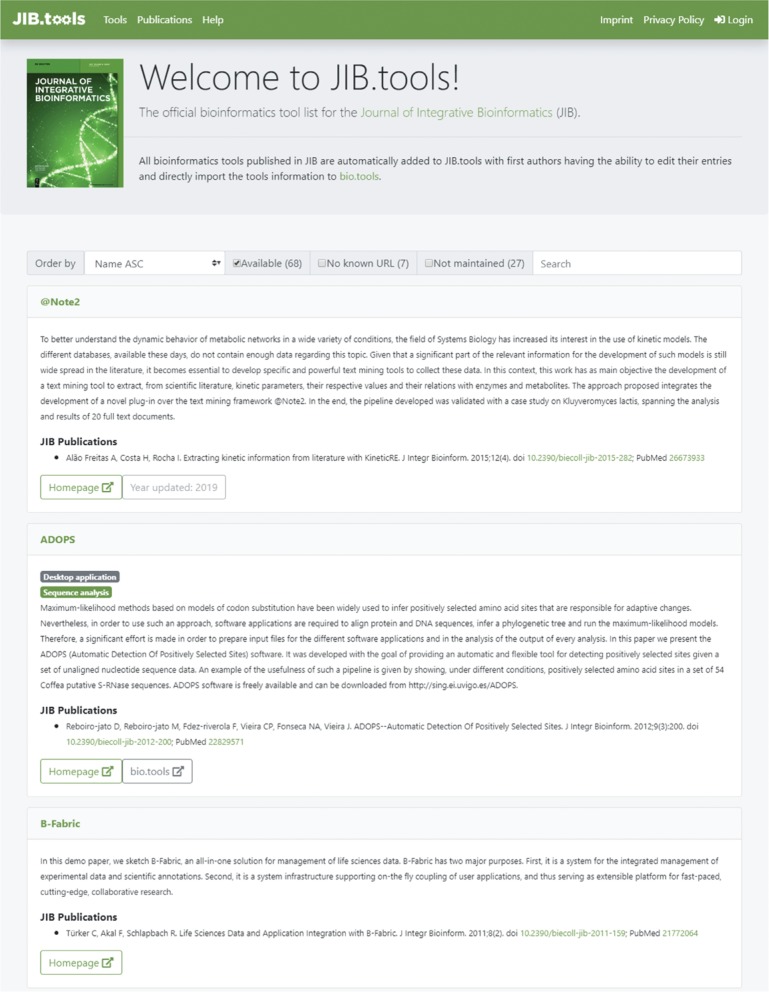
JIB.tools 2.0: the entry page of JIB.tools providing different filtering options and a search field.

A key task in maintaining a registry is to regularly add new entries and update old ones. As JIB.tools 2.0 exclusively focuses on JIB-published tools, new publications can be automatically ingested from PubMed using the query term “J Integr Bioinform [jour]”. Publication entities are populated from PubMed data, such as title, abstract, publication date, PubMed ID, and digital object identifier (DOI) using the public PubMed application programming interface (API). An AMA style citation is programmatically generated by the ingest script. The entry webpage of JIB.tools is shown in Figure 4.

The curation of data in JIB.tools is primarily supervised by the journal editors. Additionally, a simple user management system in JIB.tools 2.0 allows willing authors to curate tool entries of their respective publications. Creation and modification of tool entries, linking of publications and the user management are directly implemented in the JIB.tools 2.0 website after login. New user are currently only created by JIB editors and there is no manual registration process.

In case a new manuscript is published by JIB, the Journal editors will decide if the content is relevant in terms of providing a new tool, database or workflow. If so, the new publication ingested from PubMed will be marked as a tool entry. Then, this entry will automatically be visible to visitors of JIB.tools. After this entry is online, the authors of the tool will be contacted by the Journal Editors, providing a link and full editing rights. In addition, the entry will be directly transferred to bio.tools, giving the author the convenient option to publish their tools in both resources.

The website mainly consists of two registry lists, one for tool entries and one for publications. Simple sorting and filter mechanisms are available in order to quickly find tools by certain criteria. A search box allows for even quicker access in case the tool’s name is already known to the user. Creating and editing tool entries is supported by two mechanisms. New tool entries are pre-populated by the publications abstract text as the description which are publicly available under the Creative Commons Attribution 4.0 International (CC BY 4.0) license. If a tool is already published in the bio.tools registry, the tool ID can be added to the metadata for cross referencing. Additionally, a mechanism can be activated, to automatically fetch the tool topics and types from the public bio.tools API for the entered tool ID. These mechanisms allow for an easier and quick curation process of tools.

To implement a bilateral data exchange between JIB.tools and bio.tools all tool entries can be exported to json format by users with editing rights to a tool. Curators on bio.tools have the possibility to automatically populate all metadata for an entry by information encoded in json. This enables the completely automatic import of a JIB.tools entry into bio.tools. A sample entry for a json-encoded JIB tool is shown in Listing 1.

**Listing: 1:** Example of the json encoded ADOPS JIB tool exported using the JIB.tools website.

## 4 Discussion

While multiple successful bioinformatics tools and visualization registries exist and superseded the idea of JIBtools 1.0, the new iteration of JIBtools provides a new angle to the visibility of tools. Registries like bio.tools have the advantage of association with a larger network and the willingness of tool creators to maintain the entries. JIB.tools 2.0 tries to compensate this with the closeness to the publication process in JIB and the immediate knowledge of newly published tools. This reduces the overall workload which can in turn be used to maintain old entries with new information. For this, the hope is to present JIB.tools as an incentive for authors to publish their tools in JIB and immediately gain access to their JIB.tools entry and being able to curate it themselves.

Currently JIB.tools is focused on interoperability with the bio.tools registry with screenshots being the only point of difference. This of course may also slow down the possibility for future custom tool metadata being implemented. On the other hand, this may help keeping the registry concise and easier to maintain in the future and new features being deliberately implemented.

In this way, JIB.tools comes with the following advantages:

• Advantages for Authors
  – Visibility on JIB.tools (focused community) as well as bio.tools (large community)
  – Easy editing of entries
  – Simple and high availability
• Advantages for Editors
  – Comprehensive overview of tools published in the journal
  – Easy curation
  – Stand-alone implementation
  – Direct link between JIB.tools and bio.tools
  – Web design can follow corporate identity of the original journal

## 5 Conclusion and Outlook

In the future we will consider supporting other external websites by providing either a link or direct export to those websites. This will however depend on the communication with the curators. The current state of JIB.tools represents a complete collection of all JIB published bioinformatics tools and easy-to-use tools for the curation and update of the registry.

First authors of existing entries in JIB.tools are invited to contact the JIB.tools editors for access to their respective tools. For future publications the intention is to contact first authors directly with the possibility to curate their newly-added tool entries.

## 6 Availability and Requirements

JIB.tools is available at https://jib.tools. To fully access all features, an up-to-date browser version should first be installed on your PC or mobile device.
