# Supplementary material for: JIB.tools 2.0 – A Bioinformatics Registry for Journal Published Tools with Interoperability to bio.tools
Source: J Integr Bioinform. 2020 Jan 8;16(4):20190059. doi: 10.1515/jib-2019-0059 (PMC7074141; doi:10.1515/jib-2019-0059)
Supplement: Supplementary file 1 [file jib-16-20190059-s001.pdf]

# 1 Supplement

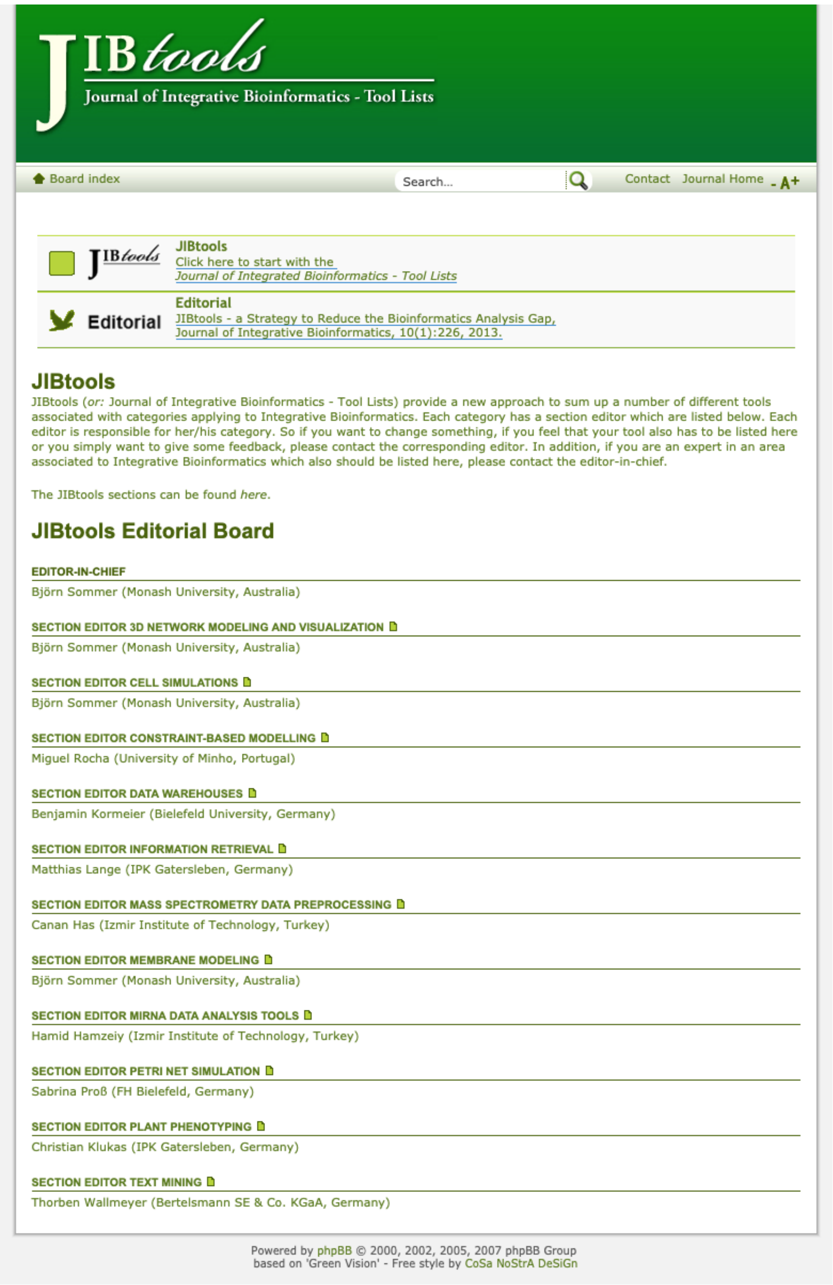


**Figure S1: JIBtools 1.0: the front page showing the different tool sections and the responsible curators**

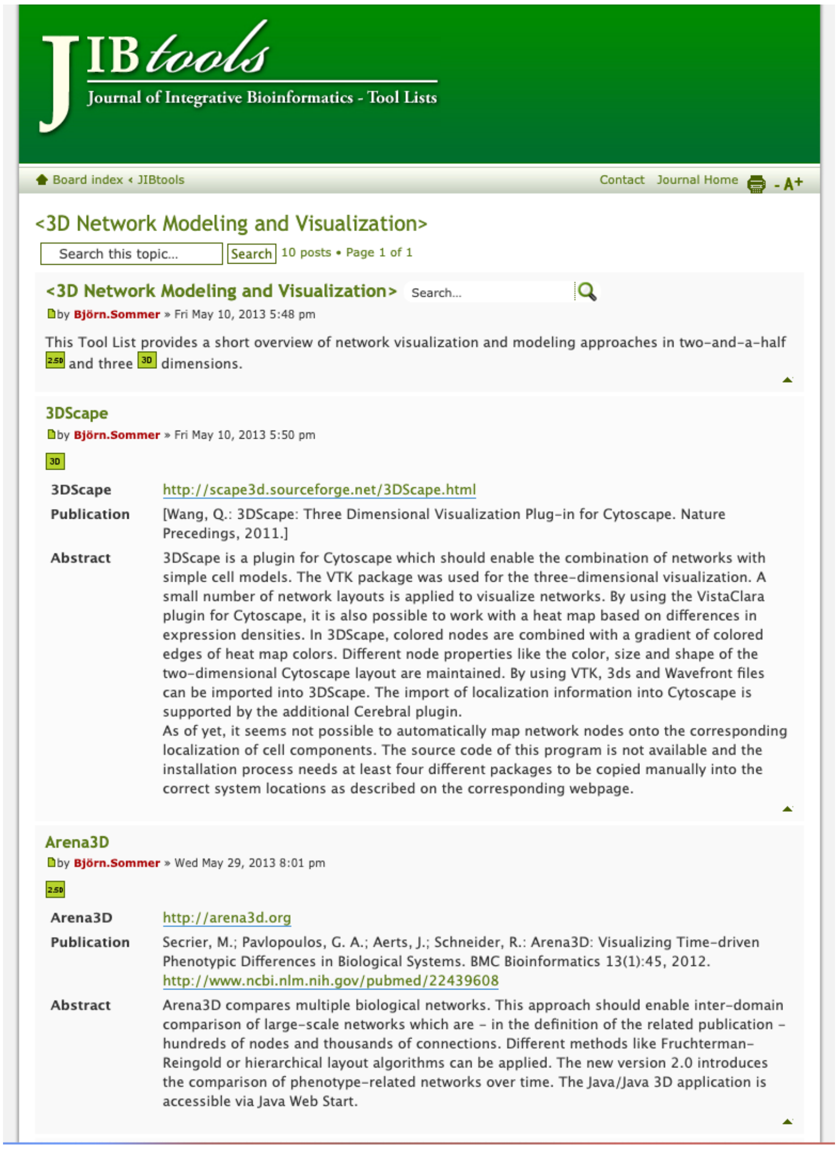

JIBtools
Journal of Integrative Bioinformatics - Tool Lists

Board index ‹ JIBtools    Contact  Journal Home  - A+

<3D Network Modeling and Visualization>

Search this topic... Search 10 posts • Page 1 of 1

<3D Network Modeling and Visualization> Search...

by Björn.Sommer » Fri May 10, 2013 5:48 pm

This Tool List provides a short overview of network visualization and modeling approaches in two-and-a-half 2.5D and three 3D dimensions.

3DScape

by Björn.Sommer » Fri May 10, 2013 5:50 pm

3D

| | |
|---|---|
| 3DScape | http://scape3d.sourceforge.net/3DScape.html |
| Publication | [Wang, Q.: 3DScape: Three Dimensional Visualization Plug-in for Cytoscape. Nature Precedings, 2011.] |
| Abstract | 3DScape is a plugin for Cytoscape which should enable the combination of networks with simple cell models. The VTK package was used for the three-dimensional visualization. A small number of network layouts is applied to visualize networks. By using the VistaClara plugin for Cytoscape, it is also possible to work with a heat map based on differences in expression densities. In 3DScape, colored nodes are combined with a gradient of colored edges of heat map colors. Different node properties like the color, size and shape of the two-dimensional Cytoscape layout are maintained. By using VTK, 3ds and Wavefront files can be imported into 3DScape. The import of localization information into Cytoscape is supported by the additional Cerebral plugin.<br>As of yet, it seems not possible to automatically map network nodes onto the corresponding localization of cell components. The source code of this program is not available and the installation process needs at least four different packages to be copied manually into the correct system locations as described on the corresponding webpage. |

Arena3D

by Björn.Sommer » Wed May 29, 2013 8:01 pm

2.5D

| | |
|---|---|
| Arena3D | http://arena3d.org |
| Publication | Secrier, M.; Pavlopoulos, G. A.; Aerts, J.; Schneider, R.: Arena3D: Visualizing Time-driven Phenotypic Differences in Biological Systems. BMC Bioinformatics 13(1):45, 2012. http://www.ncbi.nlm.nih.gov/pubmed/22439608 |
| Abstract | Arena3D compares multiple biological networks. This approach should enable inter-domain comparison of large-scale networks which are – in the definition of the related publication – hundreds of nodes and thousands of connections. Different methods like Fruchterman-Reingold or hierarchical layout algorithms can be applied. The new version 2.0 introduces the comparison of phenotype-related networks over time. The Java/Java 3D application is accessible via Java Web Start. |

**Figure S2: JIBtools 1.0: the tool section for the topic “3D Network Modeling and Vizualization”. It shows the use of different categories; here, 3D and 2.5D. Each tool comes with the link, the regarding publication and the abstract. Both tools shown here were never published in JIB. However, the list contains tools published in JIB.**
